# Scavenging of nitric oxide up-regulates photosynthesis under drought in *Festuca arundinacea* and *F. glaucescens* but reduces their drought tolerance

**DOI:** 10.1038/s41598-022-10299-5

**Published:** 2022-04-20

**Authors:** Dawid Perlikowski, Katarzyna Lechowicz, Izabela Pawłowicz, Magdalena Arasimowicz-Jelonek, Arkadiusz Kosmala

**Affiliations:** 1 Plant Physiology Team, Institute of Plant Genetics, Polish Academy of Sciences, 60-479 Poznan, Poland; 2 Department of Plant Ecophysiology, Faculty of Biology, Institute of Experimental Biology, Adam Mickiewicz University, 61-614 Poznan, Poland

**Keywords:** Physiology, Plant sciences

## Abstract

Nitric oxide (NO) has been proven to be involved in the regulation of many physiological processes in plants. Though the contribution of NO in plant response to drought has been demonstrated in numerous studies, this phenomenon remains still not fully recognized. The research presented here was performed to decipher the role of NO metabolism in drought tolerance and the ability to recover after stress cessation in two closely related species of forage grasses, important for agriculture in European temperate regions: *Festuca arundinacea* and *F. glaucescens*. In both species, two genotypes with distinct levels of drought tolerance were selected to compare their physiological reactions to simulated water deficit and further re-watering, combined with a simultaneous application of NO scavenger, 2-phenyl-4,4,5,5-tetramethylimidazoline-1-oxyl-3-oxide (PTIO). The results clearly indicated a strong relationship between scavenging of NO in leaves and physiological response of both analyzed grass species to water deficit and re-watering. It was revealed that NO generated under drought was mainly located in mesophyll cells. In plants with reduced NO level a higher photosynthetic capacity and delay in stomatal closure under drought, were observed. Moreover, NO scavenging resulted also in the increased membrane permeability and higher accumulation of ROS in cells of analyzed plants both under drought and re-watering. This phenomena indicate that lower NO level might reduce drought tolerance and the ability of *F. arundinacea* and *F. glaucescens* to recover after stress cessation.

## Introduction

Water deficit is one of the most significant environmental stresses disturbing plant metabolism and reducing their productivity worldwide^1,2^. However, plants have developed multiple strategies to cope with these stress conditions. The enhancement of metabolic pathways to cope with short events of drought and to maintain cellular homeostasis involves a decrease of water transpiration, synthesis of osmoprotectants, up-regulation of antioxidant system to scavenge reactive oxygen species (ROS), and rearrangement of cellular membranes. On the other hand, the modifications of plant organs to deal with prolonged events of drought involve reduction of leaf growth and elongation, enhanced development of roots and storage tissues. Irrespective of facing either short or long term drought, the essential part of plant response to these stress conditions is a rapid and proper stress signaling to reduce water loss, which is achieved mainly by fast stomatal closure^3^, the process regulated by plant hormones.

Under drought conditions, abscisic acid (ABA) plays the essential role in sensing the environmental changes and provides the stress signaling from the roots to the upper parts of plant organism in order to prepare plants for water shortage periods. Moreover, the mechanism of its action requires a production of hydrogen peroxide (H_2_O_2_) which is necessary for the activation of Ca^2+^ channels^4^. On the other hand, H_2_O_2_ is also classified into the group of ROS which in higher amounts become highly toxic for plant cells, leading to lipid peroxidation and membrane damage^5^. While ABA content increases in leaves, the stomatal conductance is reduced and stomata are closed, at least partially^6^. A reduction of transpiration is one of the earliest responses in plants to water deficit. Its main physiological role is to keep the cellular water potential at the necessary minimal level which is required for metabolic reactions^7^. However, stomatal closure has also negative effects on plant metabolism, which is the limitation of CO_2_ influx into mesophyll structures, leading to the inhibition of photosynthesis^8^. Furthermore, a restrained assimilation rate of CO_2_ in the Calvin cycle ends up also in the generation of ROS. Over-accumulation of NADPH and deficiency of NADP^+^ in chloroplasts significantly reduce the retake of electrons from electron-transport chain (ETC). Overloaded ETC transfers the electrons from ferredoxin to O_2_ molecule, leading to the generation of superoxide radicals (O_2_^·−^)^9,10^.

Recent studies demonstrated that ABA functioning might be regulated at many levels by the other cellular compounds. One of them is nitric oxide (NO) which is believed to modulate plants’ response to stress conditions, depending on the level of its production^11^. NO is a small gaseous molecule present in plant cells also under optimal environmental conditions. Its small size and low polarity allows it to diffuse through membranes^12^. However, its production temporarily fluctuates under both biotic and abiotic stimuli^13,14^. In plant tissues, this particle can play a double function. As one of reactive nitrogen species (RNS)^15^ it can function also as a signaling molecule^12^. However, over-production of NO can lead to the nitrosative stress in plant cells. NO reacts with O_2_^·−^ and peroxynitrite (ONOO^−^) and nitrogen dioxide (NO_2_), are generated. The over-accumulated RNS might lead further to redox-based modifications of proteins. NO itself can react with cysteine leading to the production of S-nitrothiols (SNOs), affecting functions of different proteins, in the process of protein S-nitrosylation^16^. On the other hand, ONOO^−^ and NO_2_ can nitrate tyrosine residues. The amount of proteins containing nitrotyrosine, in turn, is a well-defined indicator of nitrosative stress intensity in cells^17^. Moreover, NO can also interact with other ROS compounds in many different ways. It is known that H_2_O_2_ is a very important signaling molecule, which in cooperation with other compounds, such as NO, can regulate plant metabolism. Furthermore, its production can also be promoted by NO^18^. Both particles can be associated with a regulation of osmolytes content, ABA-induced activity of antioxidant system, stomatal movement and improve photosynthesis^19–22^. NO under stress conditions might act also as ROS scavenger which minimizes their harmful effects on plant metabolism^23^. The application of NO donors might reduce contents of malondialdehyde (MDA), O_2_^·−^ and H_2_O_2_ in plant cells under drought, enhancing the enzymatic or non-enzymatic antioxidant system^24–26^.

A regulatory function of NO is mainly associated with plants’ hormonal system. NO acts as a secondary messenger for most of plant hormones, such as cytokinins, auxins, ethylene, jasmonic acid (JA) and ABA, modulating their functions in plant development or stress responses^27–29^. Recent findings suggest the important role of NO in multiple physiological processes, including seed germination, flowering or root development^30,31^. Thus it is not surprising that ABA, which is the main stress responsive plant hormone, might be also highly affected by NO, especially when both compounds are produced under stress conditions. However, the precise role of NO in the modulation of ABA functions is still under the intensive studies, since the research outcomes obtained by different scientists, are divergent^12^. Several studies investigating the role of exogenous NO, in the form of NO donors, such as sodium nitroprusside (SNP) or nitrosoglutathione (GSNO), or the role of endogenous NO, after application of NO scavengers, such as 2-4-carboxyphenyl-4,4,5,5-tetramethylimidazoline-1-oxyl-3-oxide (cPTIO), indicated a significant role of NO generation in the induction of stomatal response under drought in the presence of ABA^32–35^. The same phenomenon was noticed also in the research on NO biosynthetic pathways involving nitrate reductase (NR) and arginine-dependent route with nitric oxide synthase-like activity (NOS)^36–38^. On the other hand, the other authors contradicted this theory, indicating that NO is not required to regulate stomatal aperture in response to ABA^11,39^. Moreover, some authors even postulated that NO can play a role of ABA negative regulator^40^. The roles of other phytohormones in the interaction with NO under stress conditions have been also studied and discussed. Most of major plant hormones were recognized to act synergistically with NO. For instance, cytokinins were previously identified to interact with NO by increasing its content during adaptation of photosynthesis to drought stress in *Zea mays*^41^. Most of the recent studies indicated that over-production of auxins or their application can significantly increase the generation of NO^29,42^. Moreover, NO itself can modulate auxins level by interacting with auxins transport and degradation processes under drought conditions^29,43^. On the other hand, the antagonistic interaction between NO and other plant hormones was also observed. Such the example is ethylene. Its biosynthesis was shown to be disrupted after application of NO^28,44^. Moreover, NO interacts also with JA, which was recognized to enhance the synthesis of NO in the guard cells, promoting stomatal closure^45^. Additionally, the application of NO can induce the expression of JA biosynthesis genes^46^. The interaction between JA and NO can play the significant role under drought treatment. It was previously observed that the application of JA to *Triticum aestivum* under drought enhanced production of NO and up-regulated the activity of ascorbate peroxidase (APX) and glutathione reductase (GR)^47^.

Though the contribution of NO in plant response to drought conditions has been demonstrated in numerous studies, this phenomenon remains still not fully recognized and requires further, more detailed analysis. For this purpose, we focused our research on forage grass species important for agriculture in European temperate regions, *Festuca arundinacea* (Fa) and *F. glaucescens* (Fg). Both species are considered as drought tolerant, however, their response to water deficit differs (e.g.^48,49^). *Festuca arundinacea* is known mainly for its ability to avoid drought by developing of deep root system, while the survival strategy of *F. glaucescens* is rather associated with an abortion of leaf metabolism under drought and its further fast regeneration in new leaves grown from the crown tissue, after stress cessation. On the other hand, the selection of genotypes distinct in their levels of drought tolerance is possible within each species. Thus, here, high drought tolerant (HDT) and low drought tolerant (LDT) genotypes of *F. arundinacea* and *F. glaucescens* were selected and used in our comprehensive research to recognize the role of endogenous NO metabolism in drought tolerance and regeneration ability of forage grasses. To do it precisely, the reactions of the selected *Festuca* genotypes to drought and re-watering conditions at physiological and molecular levels were analyzed in the presence of 2-phenyl-4,4,5,5-tetramethylimidazoline-1-oxyl-3-oxide (PTIO), which is well-known NO scavenger. We hypothesize that scavenging of endogenous NO can have a high impact on the physiological processes, such as photosynthesis and antioxidant activity under water deficit and further re-watering conditions, and simultaneously can induce alterations in drought tolerance and the ability to recover after stress cessation in *F. arundinacea* and *F. glaucescens*.

## Results

### NO generation

#### *F. arundinacea*

**Figure 1:**
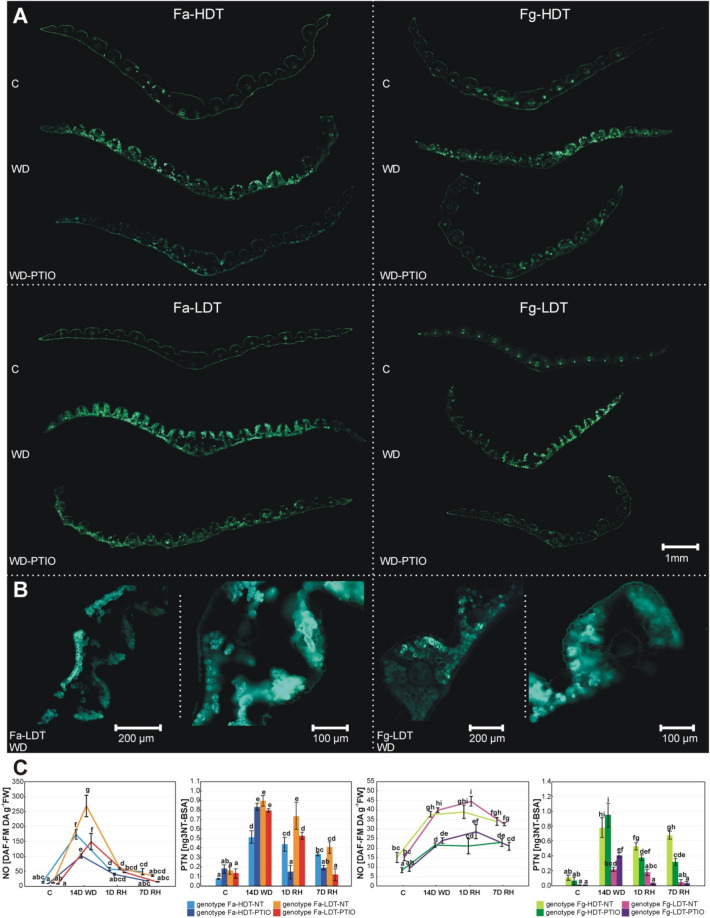
Representative bio-images of nitric oxide (NO) level in whole leaf cross sections (**A**) at the control conditions without PTIO (C), on 14th day of water deficit without PTIO (WD) and on 14th day of water deficit with PTIO (WD-PTIO). Bio-imaging of NO level in the selected magnified leaf sections of Fa-LDT and Fg-LDT genotypes (**B**). The level of NO and nitrotyrosine (PTN) (**C**), in leaves of the analyzed high drought tolerant (HDT) and low drought tolerant (LDT) *Festuca arundinacea* (Fa) and *F. glaucescens* (Fg) genotypes non-treated with PTIO (NT) and treated with 200 µM PTIO (PTIO) at control conditions (C), on the 12th day of water deficit (12D WD), 14th day of water deficit (14D WD), 1st day of re-watering (1D RH) and 7th day of re-watering (7D RH). Error bars represent the standard errors. Homogeneous groups are denoted by the same letter according to Fisher-LSD test (p = 0.05). *PTIO* 2-phenyl-4,4,5,5-tetramethylimidazoline-1-oxyl-3-oxide.

Application of drought stress drastically increased the generation of NO in both genotypes mainly in mesophyll and bundle sheath cells (Fig. 1A,B), however, Fa-LDT revealed higher NO production (Fig. 1C, Table 1). In plants treated with PTIO, reduced NO level under drought was observed, compared with non-treated plants. After 24 h of re-watering, NO level was significantly reduced in all the experimental variants. Moreover, after 7 days of re-watering all the plant experimental variants achieved the control values of NO level (Fig. 1C).

**Table 1:**
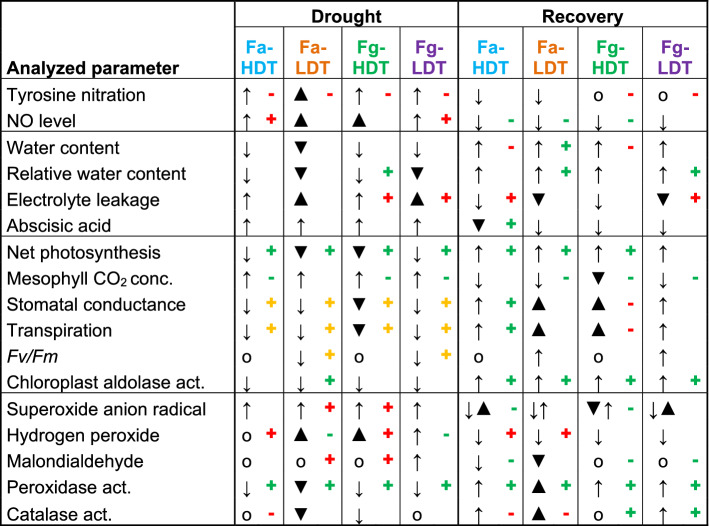
Summarized comparison of the most significant changes among analyzed parameters.

(↓) indicates the down-regulation of the parameter; (o) indicates no significant change; (bold arrows) indicates the differences between PTIO non-treated HDT and LDT genotypes; (+**)** indicates the up-regulation effect of PTIO compared to non-treated plants; (−) indicates the down-regulation effect of PTIO compared to non-treated plants; (green) indicates the positive impact of PTIO on plant functioning; (red) indicates the negative impact of PTIO; (yellow) indicates the positive impact on plant productivity but negative on drought tolerance. *Conc.* concentration, *act.* activity, *PTIO *2-phenyl-4,4,5,5-tetramethylimidazoline-1-oxyl-3-oxide, *Fv/Fm* maximum quantum efficiency of photosystem II.

#### *F. glaucescens*

Bio-localization of NO generated under drought in *F. glaucescens* genotypes was similar to that observed in Fa plants (Fig. 1A,B), however, slightly different NO generation patterns were observed between the species under the same experimental conditions (Fig. 1C, Table 1). As expected, PTIO treated plants had significantly lower NO level than non-treated plants under water deficit and re-watering time-points. However, the differences in NO level between Fg-LDT and Fg-HDT genotypes were visible only in case of PTIO treated plants and only after 24 h of re-watering, when PTIO treated Fg-LDT genotype exhibited a higher level of NO, compared to PTIO treated Fg-HDT genotype. A noticeable lower level of NO under the same conditions, compared to *F. arundinacea* genotypes, resulted from the higher auto fluorescence level of *F. glaucescens* leaves (Fig. 1C).

Moreover, during re-watering period both species reacted differentially with respect to the level of NO production. While NO level decreased significantly in *F. arundinacea* genotypes, *F. glaucescens* genotypes were characterized by its slightly elevated level (Fig. 1C, Table 1).

### Tyrosine nitration

#### *F. arundinacea*

Drought had a significant impact on the nitration of protein tyrosines. Its level increased in both plants but was higher in Fa-LDT genotype. It decreased after a full re-watering period. PTIO treatment increased nitration in Fa-HDT under drought but decreased its level after re-watering in both genotypes (Fig. 1C, Table 1).

#### *F. glaucescens*

In Fg plants non-treated with PTIO, drought increased the nitration level that was higher in Fg-HDT genotype. It decreased only after an initial stage of re-watering period and only in Fg-HDT. PTIO treatment under drought increased nitration level only in Fg-LDT, while during the advanced stage of re-watering a decrease was observed only in Fg-HDT (Fig. 1C, Table 1).

### Water relations and membrane stability

#### *F. arundinacea*

**Figure 2:**
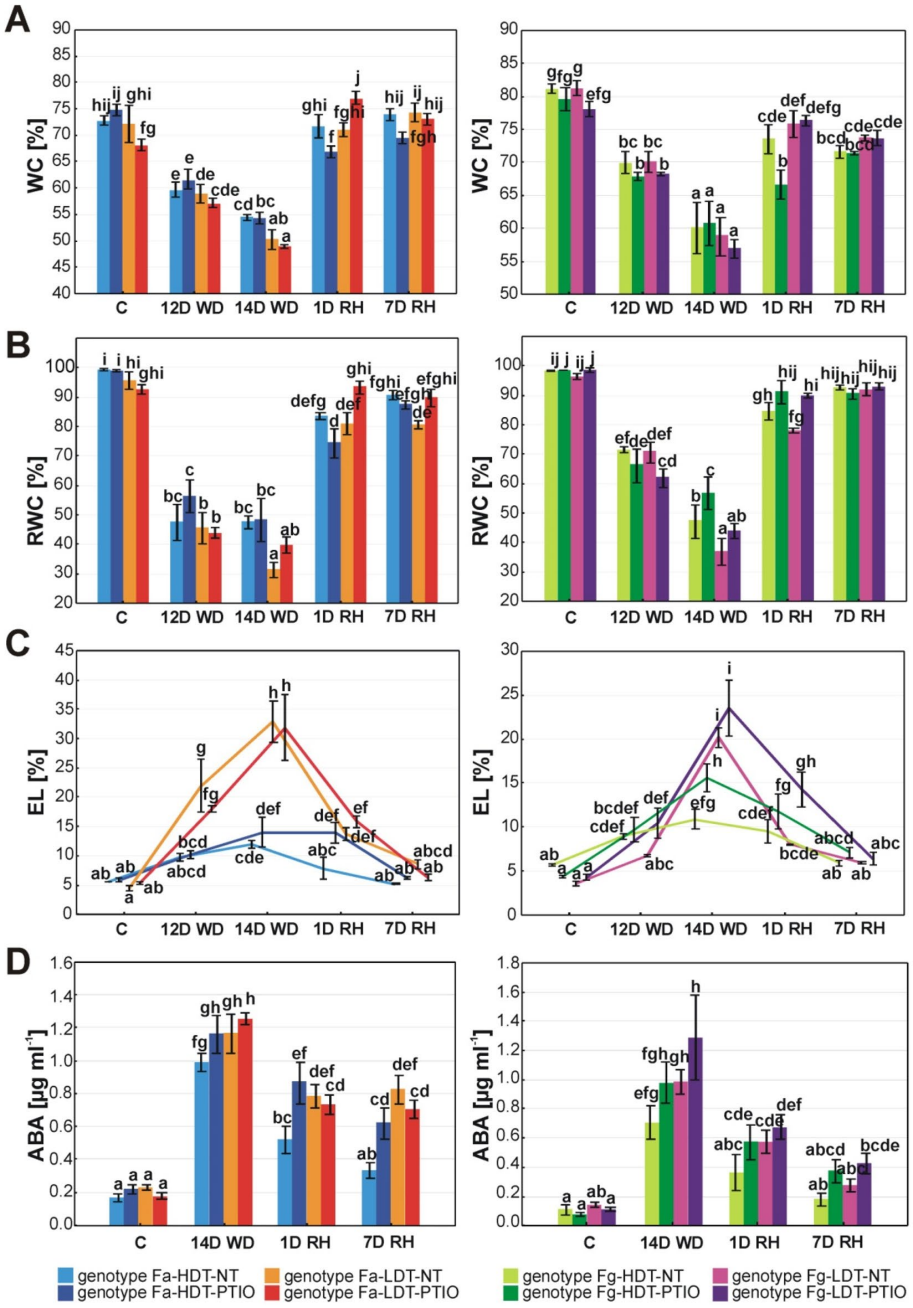
Changes in the leaf physiological parameters: water content (WC) (**A**), relative water content (RWC) (**B**), electrolyte leakage (EL) (**C**) and accumulation of abscisic acid (ABA) (**D**) in the analyzed high drought tolerant (HDT) and low drought tolerant (LDT) *Festuca arundinacea* (Fa) and *F. glaucescens* (Fg) genotypes non-treated with PTIO (NT) and treated with 200 µM PTIO (PTIO) at control conditions (C), on the 12th day of water deficit (12D WD), 14th day of water deficit (14D WD), 1st day of re-watering (1D RH) and 7th day of re-watering (7D RH). Error bars represent the standard errors. Homogeneous groups are denoted by the same letter according to Fisher-LSD test (p = 0.05). *PTIO* 2-phenyl-4,4,5,5-tetramethylimidazoline-1-oxyl-3-oxide.

Clear differences in water content (WC) between two Fa genotypes appeared after 14 days of water deficit, when Fa-HDT revealed a higher WC, compared to Fa-LDT. After re-watering, WC increased in both genotypes similarly. Application of PTIO did not have any impact on WC in case of both plants during drought treatment. However, after re-hydration, PTIO reduced WC in Fa-HDT and increased this parameter in Fa-LDT at the beginning of re-watering period (Fig. 2A, Table 1).

A decrease of relative water content (RWC) after 12 days of water deficit was noticeable in both *F. arundinacea* genotypes. However, differences between the genotypes non-treated with PTIO were visible only after 14 days of stress duration when Fa-LDT was characterized by a significantly lower RWC. After re-hydration, both genotypes regained a higher RWC. Following 7 days of re-hydration this increase was more noticeable in Fa-HDT genotype, while the genotype Fa-LDT did not fully recover. Application of PTIO had a significant positive impact only in Fa-LDT, after 24 h of re-watering when this parameter reached the control values. This phenomenon was not observed in non-treated Fa-LDT plants (Fig. 2B, Table 1).

Electrolyte leakage (EL) increased significantly under drought conditions already after 12 days of water deficit in Fa-LDT non-treated with PTIO, and after 14 days, in Fa-HDT but this genotype was characterized by significantly lower membrane damage. EL decreased after re-watering, reaching the control values after 7 days of re-hydration in both genotypes. Application of PTIO did not influence significantly EL parameter under drought conditions in Fa-LDT and Fa-HDT. Only a significant reduction of membrane regeneration rate at the initial time-point of re-hydration in Fa-HDT, was noticeable (Fig. 2C, Table 1).

#### *F. glaucescens*

In case of *F. glaucescens* genotypes WC decreased gradually under drought in both genotypes. However, after re-watering, WC of both plants increased, but did not reach the control values. Application of PTIO significantly reduced a recovery of WC at the initial stage of re-hydration in Fg-HDT, compared to the non-treated plants (Fig. 2A, Table 1).

Compared to Fa genotypes, for Fg plants we observed a more gradual decrease in RWC parameter, comparing the control, earlier and later phase of drought. The analyzed genotypes differentiated significantly after 14 days of drought. At this time-point, the genotype Fg-LDT was characterized by a significantly lower RWC. Both genotypes fully recovered RWC after 7 days of re-hydration. Application of PTIO increased the level of RWC on the last day of drought treatment only in Fg-HDT genotype. Also the plants treated with PTIO recovered RWC faster, reaching the control values after 24 h of re-hydration (Fig. 2B).

We could observe a statistically significant increase of EL parameter after 12 days of drought in Fg-HDT genotype, but the genotype dependent differences started to be noticeable after last day of water deficit when the highest EL values were reached by Fg-LDT genotype. However, after initiation of re-watering Fg-LDT genotype significantly reduced EL. Full recovery with respect to EL was reached after 7 days of re-watering in both genotypes. PTIO treatment had a visible impact on both genotypes, increasing their EL during the experiment. However, the observed changes were statistically significant for the genotype Fg-LDT only at the earlier stage of drought and at the initial stage of re-watering, while for Fg-HDT genotype only on last day of drought duration (Fig. 2C, Table 1).

Comparing two genotypes distinct in their levels of drought tolerance within and between *Festuca* species, with respect to the analyzed physiological parameters, some clear differences could be observed. First of all, a significantly lower level of WC was observed in Fa-LDT but not in Fg-LDT genotype under the later stage of drought, compared with their HDT counterparts. On the other hand, RWC was lower in both LDL genotypes in these conditions. Furthermore, a treatment with PTIO increased RWC only in Fg-HDT. *F. arundinacea* was characterized by a faster water loss at the earlier stage of drought, with respect to RWC parameter. However, more interesting phenomenon was observed in PTIO treated plants during re-hydration. A recovery of PTIO treated HDT genotypes of both species was significantly delayed especially with respect to WC parameter, while a recovery of PTIO treated LDT plants showed the opposite effect, especially with respect to RWC parameter. Electrolyte leakage was generally higher in PTIO treated plants but at different time-points for particular genotypes and species. This phenomenon was observed in Fg-LDT at the earlier stage of drought and in Fg-HDT at the later stage of drought. On the other hand, it was observed also after re-watering period in case of Fa-HDT and Fg-LDT genotypes (Fig. 2, Table 1).

### ABA content

#### *F. arundinacea*

ABA content increased in both Fa genotypes under drought. After the initiation of re-watering, it started to decrease, and in Fa-HDT it reached the control level after 7 days of re-hydration, what was not observed in Fa-LDT. PTIO treatment increased ABA content only in Fa-HDT and only after re-watering (Fig. 2D, Table 1).

#### *F. glaucescens*

Drought also increased ABA content in Fg genotypes. After re-watering, both plants reduced this content to the values observed in the control conditions. Plant treatment with PTIO revealed a slight increase of ABA content in *F. glaucescens*, compared to non-treated plants. This higher ABA content was noticeable both in drought and re-watering periods, but not at the level of statistical significance (Fig. 2D, Table 1).

### Gas exchange and photosynthesis

#### *F. arundinacea*

**Figure 3:**
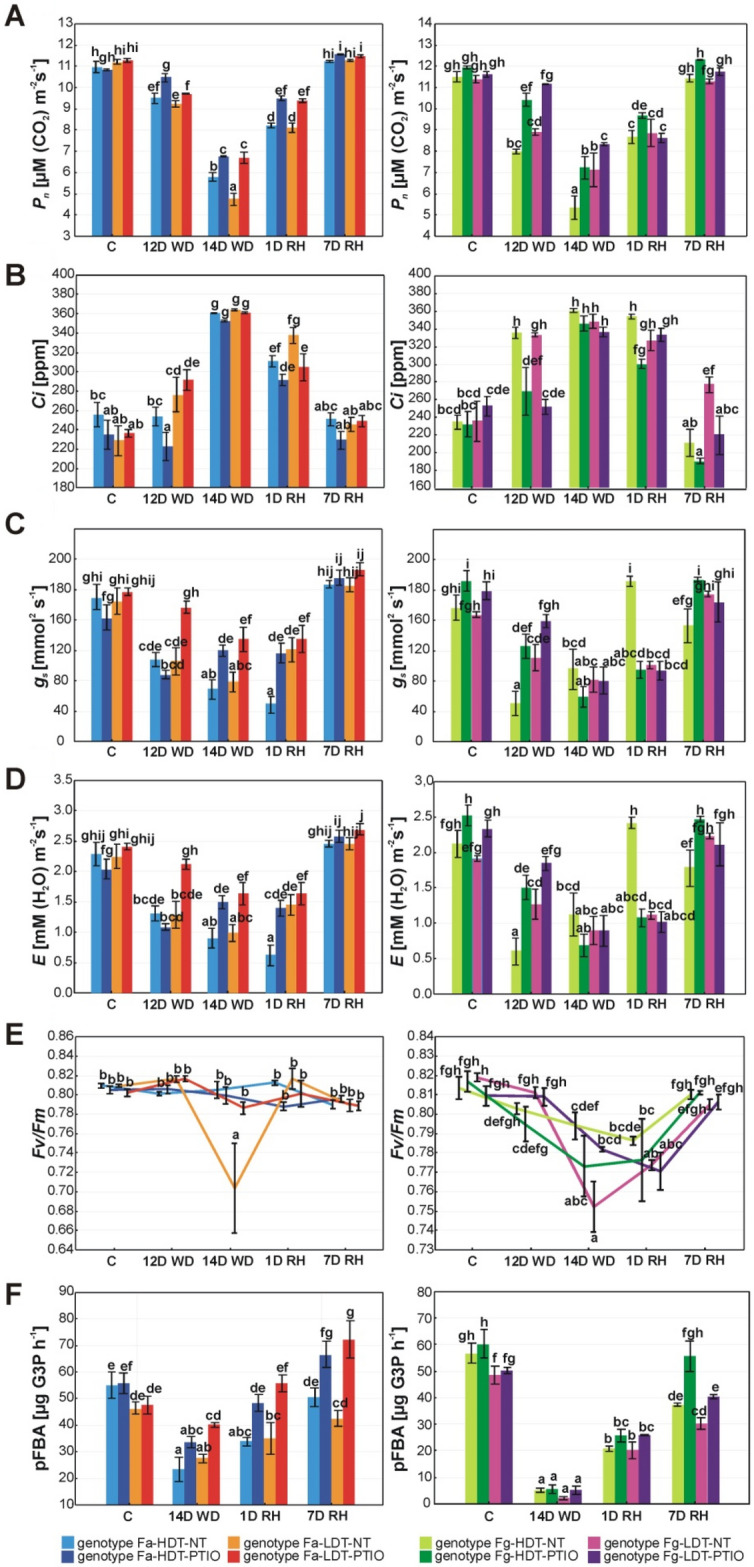
Changes in the leaf photosynthesis parameters: CO_2_ assimilation, net photosynthesis (*P*_*n*_) (**A**), internal CO_2_ concentration (*Ci*) (**B**), stomatal conductance (*g*_*s*_) (**C**), transpiration (*E*) (**D**), maximum quantum efficiency of photosystem II photochemistry (*Fv/Fm*) (**E**) and fructose-1,6-bisphosphate aldolase (pFBA) activity (**F**) in the analyzed high drought tolerant (HDT) and low drought tolerant (LDT) *Festuca arundinacea* (Fa) and *F. glaucescens* (Fg) genotypes non-treated with PTIO (NT) and treated with 200 µM PTIO (PTIO) at control conditions (C), on the 12th day of water deficit (12D WD), 14th day of water deficit (14D WD), 1st day of re-watering (1D RH) and 7th day of re-watering (7D RH). Error bars represent the standard errors. Homogeneous groups are denoted by the same letter according to Fisher-LSD test (p = 0.05). *PTIO* - 2-phenyl-4,4,5,5-tetramethylimidazoline-1-oxyl-3-oxide.

CO_2_ assimilation rate (*P*_*n*_) started to decrease in Fa genotypes after 12 days of drought and reached the lowest values after 14 days of stress duration with a significantly higher level observed in Fa-HDT. Both genotypes recovered *P*_*n*_ to the similar levels during re-watering. PTIO treated plants were characterized by significantly higher *P*_*n*_ under drought and at the beginning of re-watering (Fig. 3A, Table 1).

Intracellular concentration of CO_2_ (*Ci*) content increased significantly in the genotype Fa-LDT after 12 days of drought and in the genotype Fa-HDT after 14 days. A reduction of this parameter after re-watering was similar in both genotypes. PTIO treatment lowered *Ci* in Fa-HDT after 12 days of drought and in Fa-LDT after 1 day of re-watering with respect to their non-treated counterparts (Fig. 3B, Table 1).

Stomatal conductance (*g*_*s*_) and transpiration (*E*) decreased under drought similarly in both Fa genotypes but after 1 day of re-watering the faster recovery of these parameters was observed only in Fa-LDT. However, after full re-hydration in both genotypes these parameters returned to the values observed in the control conditions. Fa-LDT genotype treated with PTIO had a significantly higher level of these parameters under the whole drought treatment, while in the genotype Fa-HDT treated with PTIO higher levels of *g*_*s*_ and *E* were observed on the last day of drought duration and at the beginning of re-watering (Fig. 3C,D, Table 1).

A significant reduction of maximum quantum efficiency of PSII photochemistry (*Fv/Fm*) was visible only in Fa-LDT after 14 days of drought but this alteration was reverted after re-watering. What is interesting, Fa-LDT treated with PTIO was characterized by stable chlorophyll *a* fluorescence parameters during the whole experiment (Fig. 3E, Table 1).

The activity of chloroplast fructose-1,6-bisphosphate aldolase (pFBA) decreased significantly in both Fa genotypes under drought but returned to the level observed in the control conditions after 7 days of re-watering. What is interesting, the application of PTIO had a positive impact on aldolase activity in both genotypes, increasing its values significantly at almost all the time-points of the experiment (Fig. 3F, Table 1).

#### *F. glaucescens*

Drought had a similar effect on *P*_*n*_ in Fg genotypes as that observed in Fa genotypes, resulting with a higher level of this parameter observed in Fg-LDT. Moreover, PTIO treatment also enhanced *P*_*n*_ during drought in both Fg genotypes. After 24 h of re-watering, an increase was observed only in Fg-HDT treated with PTIO (Fig. 3A, Table 1).

*Ci* elevated significantly in both Fg plants at the beginning of water deficit and returned to the values observed in the control conditions after 7 days of re-watering but only in Fg-HDT. PTIO treatment significantly reduced the increase of *Ci* at the beginning of drought in both plants, after beginning of re-watering in Fg-HDT and after 7 days of re-watering in Fg-LDT genotype (Fig. 3B, Table 1).

A faster decrease of *g*_*s*_ and *E* was observed in Fg-HDT genotype after 12 days of drought but this genotype was also characterized by a faster recovery of these parameters after 1 day of re-watering. PTIO treatment significantly delayed g_s_ and *E* response on the 12th day of drought in both plants and after 7 days of re-watering in Fg-HDT genotype (Fig. 3C,D, Table 1).

Similarly as it was observed in Fa plants, Fg-LDT genotype was characterized by a significant reduction of *Fv/Fm* parameter after 14 days of drought and after initiation of re-watering. For Fg-HDT plant only a slight decrease in this parameter was visible and it was significant only after 1 day of re-watering. In comparison, PTIO treated plants also up-regulated *Fv/Fm* parameter after 14 days of drought in case of Fg-LDT, compared to the non-treated plants (Fig. 3E, Table 1).

The activity of pFBA decreased significantly in both analyzed Fg genotypes under drought but increased after re-watering. However, even after 7 days of re-watering the control values of activity were not achieved in case of both genotypes. On the other hand, at this time-point a significantly higher activity of pFBA in PTIO treated plants, compared with non-treated, was observed (Fig. 3F, Table 1).

*F. glaucescens* genotypes treated with PTIO had significantly slower stomatal response to drought, compared to *F. arundinacea*, as the differences between PTIO treated and non-treated plants were much more significant at the earlier stage of drought. Moreover, this phenomenon was also recognized after initiation of recovery in Fg-HDT genotype, which opened stomata and increased transpiration just after re-watering in the presence of elevated level of NO, compared to PTIO treated plants (Fig. 3).

### Reactive oxygen species and lipid peroxidation

#### *F. arundinacea*

**Figure 4:**
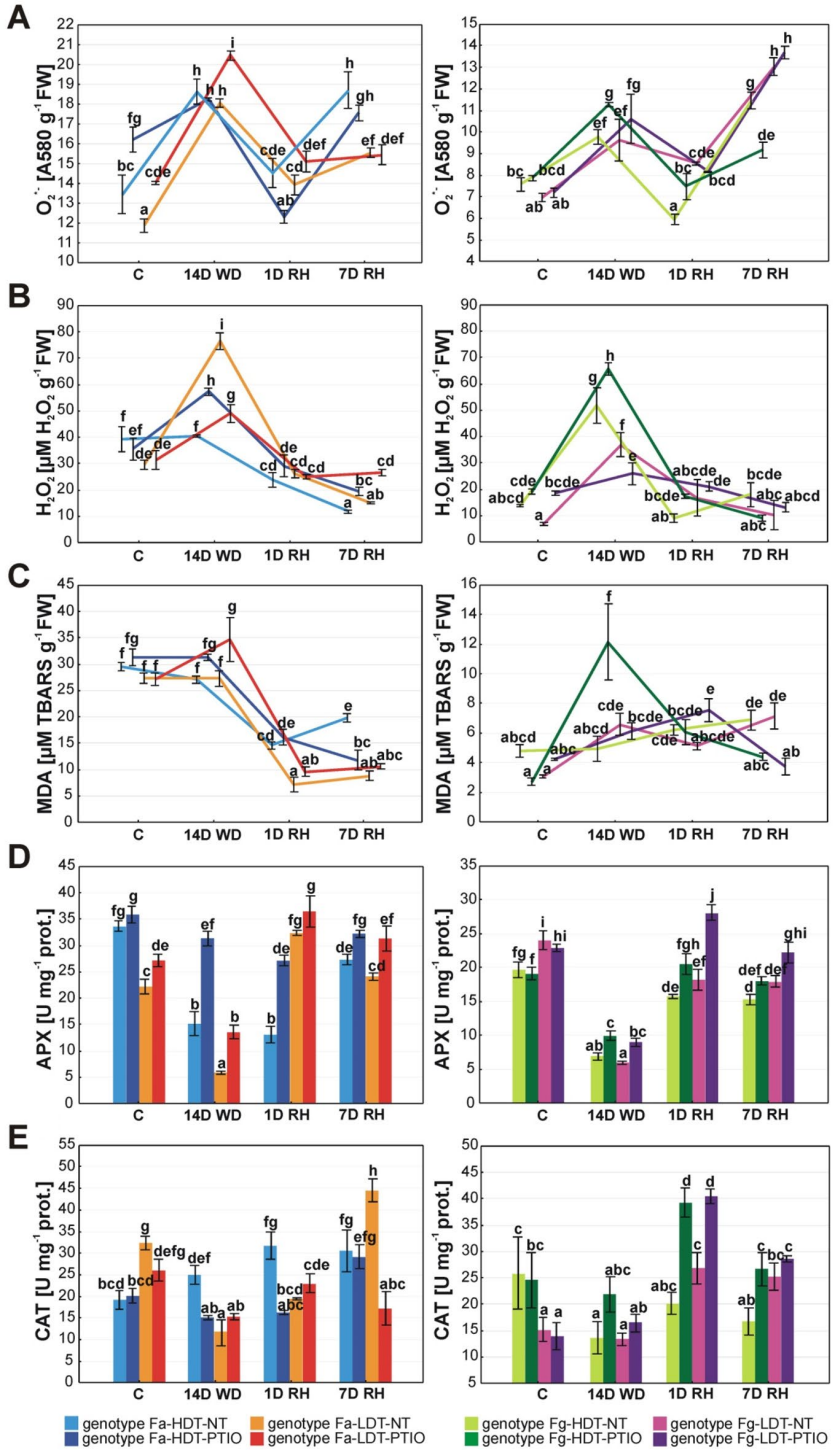
Changes in reactive oxygen species content: superoxide anion radical (O_2_^∙−^) (**A**), hydrogen peroxide (H_2_O_2_) (**B**), malondialdehyde (MDA) (**C**) and antioxidant enzymes activity: ascorbate peroxidase (APX) (**D**) and catalase (CAT) (**E**) in the analyzed high drought tolerant (HDT) and low drought tolerant (LDT) *Festuca arundinacea* (Fa) and *F. glaucescens* (Fg) genotypes non-treated with PTIO (NT) and treated with 200 µM PTIO (PTIO) at control conditions (C), on the 12th day of water deficit (12D WD), 14th day of water deficit (14D WD), 1st day of re-watering (1D RH) and 7th day of re-watering (7D RH). Error bars represent the standard errors. Homogeneous groups are denoted by the same letter according to Fisher-LSD test (p = 0.05). *PTIO* 2-phenyl-4,4,5,5-tetramethylimidazoline-1-oxyl-3-oxide.

Under water deficit conditions, O_2_^·−^ content increased in both genotypes. After 1 day of re-watering its level was reduced in both genotypes, but increased again after 7 days of re-watering period, especially in Fa-HDT. Application of PTIO increased O_2_^·−^ content in Fa-LDT under the control and drought conditions. In case of Fa-HDT genotype, PTIO increased O_2_^·−^ content in the control conditions but decreased it again in the initial stage of re-watering (Fig. 4A).

Drought increased significantly the content of H_2_O_2_ in Fa-LDT genotype but had no impact on Fa-HDT. After re-watering, both plants reduced its content in a similar way. PTIO treatment had distinct effect on H_2_O_2_ accumulation in Fa genotypes under drought. H_2_O_2_ content was higher in case of Fa-HDT and lower in Fa-LDT treated with PTIO, compared with non-treated plants. PTIO-treated plants had also higher H_2_O_2_ content after full re-watering period, compared to their non-treated counterparts (Fig. 4B, Table 1).

MDA content did not change significantly under drought in both Fa genotypes but after re-watering it was visibly reduced, especially in Fa-LDT genotype. PTIO application increased MDA content in Fa-LDT under drought and decreased it after full re-watering in Fa-HDT (Fig. 4C).

#### *F. glaucescens*

Fg genotypes were more unified with respect to O_2_^·−^ accumulation pattern. Both genotypes increased O_2_^·−^ content under drought, then reduced it at the beginning of re-watering and increased again at the end of re-watering, especially in Fg-LDT. Plants treated with PTIO presented similar patterns of O_2_^·−^ accumulation during experiment to these observed for the non-treated plants. A significantly increased content of O_2_^·−^ after PTIO application was observed only for Fg-HDT genotype under drought and after initiation of re-watering, while after full re-watering a higher content of O_2_^·−^ was observed in non-treated Fa-HDT genotype (Fig. 4A, Table 1).

Accumulation of H_2_O_2_ under drought was visible in both genotypes but its content was higher in Fg-HDT. However, that elevated amount of this particle was reduced after re-watering. PTIO had a different impact on both genotypes. In case of Fg-HDT, it increased H_2_O_2_ content under drought, while in case of Fg-LDT its level was lower, compared to the non-treated plants (Fig. 4B, Table 1).

Contrary to Fa genotypes, MDA content increased under drought and after full re-watering in Fg-LDT genotype, while it was stable in Fg-HDT. However, Fg-HDT treated with PTIO was characterized by a high increase of MDA content under drought. After full re-watering, both genotypes treated with PTIO presented lower MDA content, compared to their non-treated counterparts (Fig. 4C, Table 1).

### Antioxidant enzyme activity

#### *F. arundinacea*

Drought treatment decreased APX activity in both Fa genotypes, however, it was higher in Fa-HDT. After re-watering, Fa-LDT was characterized by a faster restoration of APX activity. PTIO treatment increased the activity of this enzyme under drought and advanced re-watering in both genotypes but following 1 day of re-watering only in Fa-HDT (Fig. 4D, Table 1).

Drought decreased catalase (CAT) activity in Fa-LDT genotype but after 7 days of re-watering its level was higher, even compared to the control conditions and compared to Fa-HDT. The Fa-HDT increased CAT activity after re-watering. PTIO treatment significantly lowered CAT activity in Fa-HDT under drought and at the beginning of re-watering. Otherwise, in case of Fa-LDT this activity was decreased by PTIO treatment after full re-watering (Fig. 4E, Table 1).

#### *F. glaucescens*

The activity of APX decreased under drought but recovered after re-watering in both Fg genotypes. The activity of this enzyme increased after PTIO treatment under drought and initial re-watering in both genotypes but after full re-watering only in Fg-LDT (Fig. 4D, Table 1).

The activity of CAT decreased under drought in Fg-HDT and remained at this level to the end of the experiment. Meanwhile, Fg-LDT increased CAT activity but only after re-watering. Contrary to Fa genotypes, PTIO treatment increased CAT activity after 1 day of re-watering in both genotypes but after following 7 days only in Fg-HDT (Fig. 4E, Table 1).

## Discussion

Up to date, several different approaches to modulate NO level in plant cells with a relation to the changing environmental conditions have been demonstrated. In most cases, exogenous NO was applied in the forms of SNP or GSNO^32,34,35,39,50^ that led to over-accumulation of NO, even in the conditions which generated a huge pool of endogenous NO. Such nitrosative conditions may therefore favor cellular homeostasis imbalance. Only a few authors presented different approaches, considering NO scavenging with the application of nitronyl nitroxides such as cPTIO or PTIO^51,52^ or application of NO in a form of gas, which does not generate secondary products^53^. In contrast to cPTIO, PTIO directly extinguishes NO without affecting NO generating sources related to the NOS-like activity^54^. Moreover, PTIO was earlier reported to be effective scavenger of NO generated during drought response of *T. aestivum* plants^52^ and in *F. arundinacea* under salt stress^55^. First attempts to decipher the role of NO generation under drought conditions suggested that this molecule is required for a positive regulation of ABA in the process of stomatal closure. In these experiments exogenous NO was applied^32,50^ or NO deficiency was induced^33^. However, the later research contradicted this theory, suggesting that NO is not required for ABA-dependent stomatal closure under water deficit^11,39^. This indicates that mechanism of NO regulation is much more complex and probably it could fulfill the dual role in modulating plant physiology^56^.

Our study to recognize the unclear role of endogenous NO in plant metabolism under drought relied on the assumption that application of PTIO to the above- (spraying) and under- (watering) ground parts of plants leads to the significant decrease of endogenous NO level. Our results clearly indicated that such the application of PTIO reduced significantly NO level under drought and re-watering periods in the mesophyll and bundle sheet cells. However, the reduction was not absolute as NO was still generated under stress conditions since PTIO does not inhibit its sources. It was also noticeable that *Festuca* genotypes characterized by lower tolerance to drought, especially Fa-LDT, generated higher amounts of NO under stress conditions, which might be associated with their higher sensitiveness to water deficit. Moreover, the observed more or less stable levels of NO during drought and re-watering periods in case of *F. glaucescens* genotypes might be associated with different strategies of this species to cope with drought conditions, compared with *F. arundinacea*^49,57^.

### Nitric oxide maintains cellular stability under drought

Our results showed that without PTIO treatment both groups of analyzed here plants, Fa and Fg, were characterized by a similar physiological response under the applied experimental conditions, as described earlier by Lechowicz et al.^49^. However, PTIO application under drought conditions and further re-watering had several significant effects on the physiology and metabolism of analyzed plants.

It was previously indicated that higher NO production helped maintaining water status in *Nicotiana tabacum* and *T. aestivum* under osmotic stress^58^ or even in *F. arundinacea* cultivars^59^, what is in contrary to our observations. Also in *T. aestivum*, application of NO led to the decrease in water consumption under polyethylene glycol (PEG) treatment which was reverted after cPTIO application^60^. On the other hand, a negative effect of PTIO treatment, which was observed only in *F. glaucescens* with respect to changes in EL parameter, might indicate that *F. glaucescens* is more sensitive to NO fluctuations, at least with respect to the stability of its cellular membranes. A similar negative effect of NO deficiency was observed earlier in *F. arundinacea* cultivars, which were characterized by lower EL under drought and salt stress in the presence of higher NO levels^55,59^. Moreover, PTIO treated Fg-HDT was also characterized by increased MDA level under drought. The previous findings indicated that a decrease of membrane damage followed by a lower level of MDA was observed in NO-treated plants under osmotic stress^61^. We suggest that this phenomenon might be associated with a higher water usage in PTIO treated plants resulted from a higher transpiration rate and increased stomatal conductance observed at the earlier stage of drought in Fa-LDT and in both Fg genotypes and after 14 days of water deficit—in both Fa genotypes. Thus, an artificially lowered level of NO observed under water deficit significantly influenced stomatal aperture and increased the level of transpiration in both *Festuca* species, making them more sensitive to dehydration at least at the earlier stage of drought. This phenomenon was supported by the other analysis which confirmed the hypothesis that the application of NO supplied as SNP donors induced stomatal closure^32^ and cPTIO application was able to revert this mechanism in the presence of ABA^51^. Moreover, higher EL observed after initiation of re-hydration, mostly in Fa-HDT, indicates that NO scavenging might significantly reduce the ability to recover after stress cessation in the analyzed plants.

### Nitric oxide can be responsible for early stomatal closure and down-regulation of photosynthesis under drought in forage grasses

A reduction of transpiration rate caused by ABA-dependent stomatal closure is the earliest response of plants subjected to water deficit conditions. This process significantly reduces the stomatal conductance in order to keep a high water potential in leaves to avoid dehydration process^7^. However, decreasing stomatal conductance simultaneously limits CO_2_ diffusion into the mesophyll, reducing CO_2_ assimilation rate and the efficiency of photosynthesis^8^. Our findings indicate that more opened stomata under PTIO treatment are not in full correlation with the observed higher content of ABA, which should be responsible for stomata control under drought^62^. It is known that this signaling pathway involves the participation of several other compounds besides ABA, such as Ca^2+^, NO, and H_2_O_2_, protein kinases and transcription factors. It has been established that NO production is required for ABA-induced stomatal closure. Moreover, this phenomenon is caused by NO accumulation in guard cells in response to ABA^63^. It has been reported before that the activity of enzymes involved in NO generation such NR or nitrate-NO oxidoreductase (NI-NOR) were enhanced by ABA^62,64^. This was supported by our observations pointing out that the increased ABA accumulation under drought was associated with a simultaneous increase of NO production in leaf tissues non-treated with the scavenger. Moreover, it was previously recognized that elevated NADPH in *Z mays* leaves induced by ABA resulted in the increased generation of NO under drought^65^. However, in PTIO treated plants it was noticeable that lower NO level somehow induced the increase of ABA accumulation. In most cases, these relations were not statistically significant, except for the re-watering phase in PTIO treated Fa-HDT. This genotype with a significantly higher content of ABA, compared to non-treated Fa-HDT, simultaneously possessed more opened stomata and higher transpiration. Nevertheless, it could be possible that the increasing content of ABA under the scarcity of NO might be associated with a positive feedback mechanism, when plants try to refill a deficiency of NO by the increasing of ABA production.

NO was previously identified as a required element responsible for ABA-dependent stomatal closure in *Vicia faba* and *Pisum sativum*^51^ and ABA was shown to be essential for inducing the synthesis of NO. Moreover, the application of NO scavenger such as cPTIO significantly reduced stomatal closure in the presence of ABA^66^. This indicates that ABA might not be sufficient for a proper signaling of stress conditions, especially at the early stages of drought and a relatively elevated production of NO might be required for the fast stomatal closure. However, these results are in contrast to some previous studies which showed that NR deficient mutant presented super sensitivity for ABA treatment^67^. Moreover, other authors indicated that NO is a negative regulator of ABA^12^ and its presence stimulates stomata opening at the early stages of drought in *Glycine max*, while its absence at the advanced stage of drought cannot prevent stomatal closure^40^. Also it was noticed that a treatment with very high amounts of NO led to stomata opening even under ABA treatment^68^. Thus, it is suggested that a relatively high level of NO is responsible for stomata closure whereas exceeding this level or depletion could have the opposite effect^69^. Additionally, it is also suggested that NO can act independently from ABA and control stomata via a different pathway^53^, since no differences between Fg genotypes were visible with respect to stomatal conductance after 14 days of drought, though PTIO was supplied. However, this might also indicate that NO is necessary in plants only at the beginning of stress period for fast response to changed environmental conditions and its depletion might significantly disturb plants’ signaling leading to the delayed reaction to stress conditions.

Drought is known to inhibit plants’ growth and development due to the impaired photosynthesis^70^. It has been reported before that high concentration of NO significantly decreased the rate of photosynthesis in *Avena sativa* and *Medicago sativa* plants^71^. In our research, an inducing effect of NO scavenging on photosynthetic rate was observed both in drought and re-watering conditions in two *Festuca* species. Plants treated with NO scavenger were characterized by the increased CO_2_ assimilation rate which, at least partially, could have been associated with higher stomatal conductance in the analyzed plants. It was observed that a decrease of photosynthesis was mainly caused by stomatal closure initiated by NO activity^34^. However, an open discussion still exists if NO acts as the enhancer or inhibitor of photosynthesis, because it was also observed that NO improved the photosynthetic rate in *T. aestivum* plants under osmotic stress^60^. Nevertheless, a treatment with SNP had simultaneously a negative impact on photosynthetic enzymes in *T. aestivum* or *Phaseolus aureus*^72,73^ but also enhanced photosynthesis rate in *Lycopersicon esculentum* or *Cucumis sativus*^74,75^. More light has been shed by the latest finding in which the exogenous NO in gaseous form drastically reduced photosynthesis without the influence on stomatal conductance, revealing that the mesophyll-driven signals induced by NO are also important in this mechanism^53^. It has to be also noticed that *F. glaucescens* treated with PTIO had significantly slower stomatal response to drought, compared to *F. arundinacea*, as differences between PTIO treated and the non-treated plants were more visible in case of Fg genotypes after 12 days of drought. Moreover, this effect was also recognized after initiation of re-watering in Fg-HDT genotype, which opened stomata and increased transpiration just after re-watering in the presence of elevated level of NO. On the other hand, PTIO treated plant did not respond even under decreased ABA content. This indicates that under NO depletion the environmental sensing and signaling was disturbed, thus the increased photosynthesis due to more opened stomata could be in fact a positive effect of NO scavenging with respect to plant productivity. On the other hand, more opened stomata and increased transpiration could lead to a faster dehydration of plant’s tissue and lowered plant’s drought tolerance.

However, also the important role of NO in the modification of enzymes involved in photosynthesis cannot be excluded. A significant effect of SNP application on photosynthetic enzymes, including the inhibition of Rubisco and Rubisco activase by S-nitrosylation was previously observed^73,76^. Moreover, tyrosine nitration or S-gluthationylation was also identified as the mechanism negatively controlling the activity of pFBA and other photosynthetic enzymes under the salinity stress^77,78^. These results are similar to the observed here with respect to the activity of pFBA, which was generally higher in PTIO treated plants, indicating that lower NO level might indeed enhance the photosynthesis also through the regulation of the Calvin cycle. However, whether this mechanism could be in fact associated with protein nitration is not clear, since a lower level of tyrosine nitration was observed only after re-watering and it does not explain the higher aldolase activity under drought when a higher level of tyrosine nitration in proteins was observed in most genotypes treated with PTIO. Nevertheless, since only the level of nitro-tyrosine was measured it cannot be excluded that lower S-nitrosylation under PTIO treatment was responsible for higher pFBA activity.

Diffusion limitations of CO_2_ influx has also a huge impact on the light phase of photosynthesis and the electron transport^8^. Moreover, NO is able to bind directly to any thiol- or metal-containing proteins^35^, including PSII complex^79^. A decrease of chlorophyll parameters and an inhibition of the light phase of photosynthesis under drought conditions is a well-known phenomenon^80,81^ and it was observed in case of LDT genotypes under drought treatment. However, this effect was significantly reduced under PTIO treatment. On the other hand, the application of NO donor, such as SNP was proven to increase the chlorophyll content in *T. aestivum*^82^ and also in *F. arundinacea* cultivars^59^. However, a recent finding proved that a higher dosage of SNP^53^ or GSNO^83^ can in fact influence functioning of PSII and can inhibit its efficiency by disturbing the electron transport between plastoqinones Q_A_ and Q_B_. It was estimated that a treatment with NO donors can significantly decrease maximal quantum efficiency (*F*_*v*_*/F*_*m*_) in vivo^79,83^. It supports our findings demonstrating that both LDT genotypes and especially Fa-LDT, characterized by the highest NO level under drought, indeed had significantly disturbed *F*_*v*_*/F*_*m*_ under drought. However, it cannot be excluded that the inhibition of photosynthetic antennas might be also a mechanism of protection^84,85^ driven by NO against hyper-oxidation observed in cells of these plants. In this case, chlorophyll content stabilized by PTIO might be treated rather as a negative phenomenon. Especially that after initiation of re-watering, the plants non-treated with PTIO were able to regenerate *F*_*v*_*/F*_*m*_ very fast, while in case of PTIO treated Fg-LDT, a decrease of that parameter was observed.

All these results indicate that NO-dependent regulation of photosynthesis is not a simple process that could be explained only in terms of on/off switch mechanism.

### Genotype-dependent effect of NO scavenging promotes ROS accumulation and antioxidant system activity

Accumulation of ROS, such as O_2_^·−^ and H_2_O_2_, and the increase of lipid peroxidation, which can be described by the elevated level of MDA in plant cells, are among the main effects of plant exposure to adverse environmental conditions, including drought stress^82,86,87^. A generation of ROS may serve a double role in plant cells. First of all, it may function as the signal transductor enhancing plant response to stress but it can also interrupt the cellular redox state, thus generating oxidative stress that causes cellular damage, such as irreversible enzyme modifications, lipid peroxidation and DNA damage^5,88^. It is very difficult to distinguish these two roles of ROS, due to their overlapping events occurring in cell environment^5^. ROS and NO can react with each other to form several RNS such as ONOO_2_, NO_2_, N_2_O_3_ and other species that can modify the activity and function of multiple proteins^89^. It was noticed that NO application in the form of SNP significantly improved MDA detoxification^82^ in *T. aestivum* under drought and in *F. arundinacea* under salt stress^55^. This phenomenon corresponds partially with our results, indicating that Fa-LDT and Fg-HDT plants with lower NO production under water deficit had significantly impaired MDA scavenging. It might also, at least partially, correspond to higher membrane permeability observed in PTIO treated Fg-HDT genotype under drought. However, this effect was opposite after re-watering period, indicating that lower amounts of NO could promote ROS scavenging.

A reduced generation of NO in the analyzed plants had very distinct effects on the contents of other ROS, depending on the genotype. Accumulation of O_2_^·−^ was generally promoted after the application of PTIO under drought in Fa-LDT and in Fg-HDT, as it was previously observed under salt stress in other *F. arundinacea* cultivars^55^. This phenomenon was also well-visible at the initial stage of re-watering in Fg-HDT but it was further reverted. A boosted O_2_^·−^ formation in Fa-LDT and Fg-HDT plants with artificially depleted NO could be due to the inability to form ONOO^−^ which reflects insignificant changes in the level of protein nitration under drought. Moreover, a reduced pool of nitrated proteins noted in all the analyzed genotypes during re-watering periods can strengthen the hypothesis that a tightly balanced level of ONOO^−^ via protein tyrosine nitration could be involved in a broad spectrum of signaling and regulatory processes^90^. The early stage of re-watering significantly reduced O_2_^·−^ level, however, its content returned to level observed under drought stress or it was even higher after 7 days of re-watering period. We suggest that this phenomenon might be associated with the hypoxia or reoxygenation events during re-watering^91^. Hypoxia drastically changes soil conditions, reducing oxygen availability in soil for roots and microorganisms, leading to accumulation of many toxic metabolites and ROS^91^. Moreover, the long term drought, when plants were cut off from water and atmospheric oxygen, and subsequent fast re-watering, leading to stomatal opening, could result in the stress of reoxygenation. This could lead in turn to O_2_^·−^ accumulation in a similar way as it was observed in flooded plants^92^. However, during re-watering plants were not exposed to flooding, thus the negative effect of fast environmental change was only limited to fluctuations of O_2_^·−^ level. Moreover, in case of the analyzed plants, the initial drought tolerance had no correlation with the level of reoxygenation, since more drought tolerant *F. arundinacea* genotype and less tolerant *F. glaucescens* were characterized by a higher level of O_2_^·−^. A lower level of NO reduced this phenomenon only in case of Fg-HDT genotype.

With respect to H_2_O_2_ content, its increase after PTIO application was observed under drought in both HDT plants. This phenomenon was also indicated by other authors under salt stress^55^. Priming *T. aestivum* seedlings with NO and H_2_O_2_ resulted in improved drought tolerance and elevated accumulation of osmolytes^19^. On the other hand, in LDT genotypes of both species a decreased content of H_2_O_2_ was observed in the analyzed conditions. These differences might be associated, at least partially, with the potential of antioxidant system. Thus, a lower activity of CAT after application of PTIO under drought was visible at least in Fa-HDT. Accumulation of H_2_O_2_ in LDT plants might as well correspond to the level of NO, as it can stimulate H_2_O_2_ production^18^. Generally, it was observed that elevated H_2_O_2_ content can mediate ABA response to enhance the production of NO, by the stimulation of NR activity^93,94^. However, comparing the plants non-treated with PTIO, we also got equivocal results. The relations between NO and H_2_O_2_ content were clear in Fa plants but simultaneously the opposite effect was observed in Fg plants. It was proposed that endogenous generation of NO and H_2_O_2_ might enhance ABA-dependent drought tolerance and improve the activity of antioxidant enzymes^95^. In *Z. mays*, it was observed that H_2_O_2_ accumulation enhanced by ABA, is required for NO synthesis, which resulted in the chain reactions leading to a stimulated expression of antioxidant enzymes and to an increase of their activities^93^. On the other hand, a decrease of H_2_O_2_ content after SNP treatment was observed previously in *Discorea opposita*^96^. NO might trigger the activity of antioxidant system to regulate H_2_O_2_ content^97^. Additionally, after 7 days of re-watering, PTIO treated *F. arundinacea* genotypes were still characterized by the elevated level of H_2_O_2_. This might indicate that lower NO level could disturb the process of recovery in this species.

Plants have a very well developed ROS scavenging system including enzymatic and non-enzymatic components that efficiently neutralize the excess of generated ROS in cells^98^. The enzymatic system requires coordinated activity of several proteins such as superoxide dismutase (SOD) which neutralize O_2_^·−^, glutathione reductase (GR), APX and CAT which are stimulated under drought conditions^99^. Lots of evidence indicate on enhancing effect of exogenous NO on the enzymes of antioxidant system, such as SOD, CAT and peroxidases (POX) under osmotic stress^60,100,101^. It was shown that SNP treatment can significantly improve antioxidant system activity, including CAT, SOD and POX under the water stress^102^, also in different *F. arundinacea* cultivars^59^. However, we could notice a strong positive effect of NO scavenging on APX activity in both *Festuca* species and on CAT activity but only in Fg plants. In Fa genotypes, PTIO treatment had no effect or had rather a negative impact on CAT activity. This phenomenon was indicated previously^59^ and might be also related to the elevated level of H_2_O_2_ at the particular time-points of drought and re-watering. Seven days of re-watering were not sufficient for a full recovery of the drought treated plants as only a part of the analyzed parameters returned to the control values. There is growing evidence that NO can modulate the activity of antioxidant enzymes by nitrosylation of particular amino acids. It was observed that S-nitrosylation of Cys-32 in APX can increase plant resistance to oxidative stress^103^. On the other hand, nitration of tyrosine in APX inhibits its activity^104^, what indicates the opposite regulatory roles of NO under stress conditions which cannot be easily explained. An inhibition of APX and CAT by exogenous NO was noted in isolated *P. sativum* mitochondria, *Pelargonium sp*. leaves, *Arabidopsis* and *N. tabacum* suspension cells^105^. According to Clark et al.^106^, a temporal redox-based inhibition of CAT and APX in response to NO treatment might suggest the participation of NO molecule in fine-tune regulation of H_2_O_2_ content.

## Conclusions

The scavenging of NO in *F. arundinacea* and *F. glaucescens* allowed the recognition of NO-dependent pathways involved in drought tolerance and the ability to recover after stress cessation in these important forage grasses. LDT and HDT genotypes of *Festuca* species revealed significant differences with respect to the level of generated NO under stress conditions, indicating that less tolerant genotypes from both species accumulated higher amounts of NO. This might be a result of their higher sensitivity to changing environmental conditions. Moreover, a significant enhancement of photosynthesis in plants with reduced NO level in mesophyll tissue under drought was noticeable for the first time in forage grasses. This phenomenon could be a result, at least partially, of inhibited stomatal closure under NO deficiency observed especially at the earlier stage of drought in two *Festuca* species. It could be also associated with a higher activity of photosynthetic enzymes. A delayed response of stomatal aperture in *F. glaucescens* genotypes to reduced NO level was also followed by its slower recovery and a lower WC in Fg-HDT genotype, after the initiation of re-watering. This indicates a negative impact of NO scavenging on the time-point at which plants respond to changed environment conditions. Moreover, NO scavenging resulted in other negative effects, including higher membrane permeability and higher accumulation of ROS. These phenomena were also observed after re-watering indicating that lower NO level might reduce not only drought tolerance but also the ability of the analyzed plants to recover after stress cessation. Our results indicate that the balanced amounts of NO could maintain the homeostasis in the cellular environment, thus too high or too low levels of NO might significantly disturb the stress signaling pathways.

## Materials and methods

### The plant materials

The plant materials involved: (i) two hexaploid (2n = 6x = 42) genotypes of *Festuca arundinacea* Schreb. cv. Kord with distinct levels of drought tolerance^48,49,107^: Fa-45—high drought tolerant (HDT) and Fa-60—low drought tolerant (LDT); and (ii) two tetraploid (2n = 4x = 28) genotypes of *F. arundinacea* Schreb. subsp. *Fenas* (Lag.) *Arcang*. (referred here as *F. glaucescens*) also with distinct levels of drought tolerance^49^: Fg-16—high drought tolerant (HDT) and Fg-1—low drought tolerant (LDT). The genotypes of *F. arundinacea* were germinated from single seeds originated from the collection of Institute of Plant Genetics, Polish Academy of Sciences, created by Professor Zbigniew Zwierzykowski in 2010. Seeds of *F. glaucescens* (ABY-Bn 354-1980) derived from the collection of the Institute of Biological, Environmental and Rural Sciences (IBERS) (UK), originated from the Centre de Recherches de Lusignan, INRA (France) and donated in 1985 to IBERS. This collection at IBERS was held by the Genetic Resources Unit (Mr. Ian D. Thomas). The genotypes of *F. glaucescens* were obtained in 2014. The analysis of genomic structure was performed for both species, *F. arundinacea* and *F. glaucescens*, to precisely confirm their identity, by Dr. Joanna Majka^49^. Both species are not under the species conservation law, thus formal national legislation is not required. All the experiments performed on these plant species were in accordance with relevant institutional guidelines and regulations.

### The experimental conditions

Pre-cultivated plant clumps of each genotype were divided into singular tillers and 3 × 10 healthy tillers in a similar stage of growth were placed as 3 separate clumps (3 biological replications) to 2.5 dm3 pot with 1 kg medium, sand: peat (1:3). Each genotype was represented by 20 pots (3 clumps each) from which 4 different experimental groups were created: (i) watered plants, (ii) plants watered with PTIO application, (iii) plants subjected to drought and without PTIO application, (iv) plants subjected to drought with PTIO application. After the cloning procedure, under installation plants were daily watered and fertilized with modified Long-Ashton nutrition solution^48^ for 2 months to achieve the satisfactory level of plant development. After this period, plants were transferred to the growth chamber with following environmental conditions: 22/17 °C day/night, 16 h photoperiod, 200 µmol/m^2^/s photosynthetic photon flux density (PPFD), and air humidity 55–60%. After additional 1 week of plants’ acclimation, PTIO treatment was applied and plants treated with PTIO started to be watered with 20 ml of 200 µM PTIO daily. Additionally, leaves were daily sprayed with 2 ml of PTIO solution. It was previously estimated that PTIO in the water solution at room temperature is stable at least for 20 h^108^. Plants without PTIO application were watered and sprayed with the same amount and frequency with distilled water. After 5 days of treatment, half of the plants from each group were subjected to drought treatment and the other half were maintained with well watering (50% of soil water capacity, SWC) up to the end of the experiment. Plants subjected for water deficit were still provided with PTIO application and an exact amount of water was distributed to non-treated plants. During the experiment, the SWC was monitored by weighting and equalizing the pots each day to maintain similar SWC in each pot in drought treated groups. After 14 days of water deficit, when SWC reached 5%, the plants were re-watered to the maximum level of SWC and maintained in full watering conditions up to 7 days. Physiological analyses and plant material collection for molecular analyses were performed at four time-points of the experiment: after 12 days of water deficit (12D WD; earlier stage of drought) (only physiological parameters), after 14 days of water deficit (14D WD; later stage of drought), after 1 (1D RH; initial stage) and 7 days (7D RH; advanced stage) of re-watering.

### NO level measurement and microscopic analysis

The level of NO was measured as the fluorescence of the products generated after the reaction with 4,5-diaminofluorescein diacetate (DAF-FM DA) (Sigma) fluorescent dye. To evaluate the level of NO, 10 discs of 0.8 cm in diameter were cut from the middle section of second fully developed leaves from 3 biological replicates. Discs were covered with 1 ml incubation buffer containing 20 µM DAF-FM DA in 10 mM HEPES–KOH, pH 7.4 and incubated for 1 h in dark and washed twice with 10 mM HEPES–KOH, pH 7.4. After the final wash, samples were finely homogenized in 1 ml HEPES buffer and centrifuged 900 g at room temperature. The fluorescence was measured with 470 nm excitation and 515 nm emission wavelength, using Fluorescence Spectrophotometer F-2500 (Hitachi, Japan). The level of NO was expressed as the relative fluorescence of DAF-FM DA calculated for 1 g of fresh biomass (S/S_FW_) − (R/R_FW_), where S is a fluorescence of sample incubated with DAF-FM DA, R is a fluorescence of reference without DAF-FM DA incubation and FW is the weight of the discs.

Leaf cross-sections were prepared from the middle part of three different second but fully developed leaves. Twenty cut cross-sections, approximately 0.1–0.3 mm thick (cut with ultra sharp razor blade), were incubated with 1 ml of 20 µM DAF-FM DA in 10 mM HEPES–KOH, pH 7.4 for 1 h in dark. After incubation, the buffer was removed and cross-sections were washed twice with 10 mM HEPES–KOH, pH 7.4. Tissue sections were placed on the slide glass and observed under the AXIO Image M2 microscope (Carl Zeiss Gottingen, Germany) equipped with a motorized stage, the Colibri LED-based fluorescent light source and the AxioCam ICc5 camera. The DAF-FM DA fluorescence was excited with 470 nm blue LED and the Zeiss filter set No. 38 HE (excitation BP 470/40, beam splitter FT 495, emission BP 525/50) was used for the imaging. Obtained data was documented and analyzed with the internal ZEN2 (blue edition) software (Carl Zeiss Jena, Germany).

### Tyrosine nitration measurements

The level of 3-nitrotyrosine modified proteins was determined using Nitrotyrosine ELISA Kit (Abcam Cambridge, UK, ab113848) following the manufacturer’s instructions with slight modifications. The amount of 0.1 g of leaf tissue was homogenized in 150 µl of 50 mM Tris–HCl buffer, pH 7.5 with 1 mM PMSF. The extract was centrifuged on QIAshredder Mini Spin Column (Qiagen) at 14,000×*g* for 10 min at 4 °C and the supernatant was used for further measurements. The assay was performed as an end-point measurement at 450 nm, after termination the reaction by adding 100 ul of 1 N HCl. Results were normalized considering the total protein content in the homogenized samples and expressed as ng 3NT-BSA per µg of protein. The sample protein concentration in the extract was determined by the Bradford’s method^109^. Spectrophotometrical measurements were performed using the Synergy HTX Multi-Mode Reader (BioTek) in 3 biological replicates.

### Physiological parameters measurement

Water content (WC), relative water content (RWC), electrolyte leakage (EL), chlorophyll ‘a’ fluorescence parameters and gas exchange parameters (CO_2_ assimilation, transpiration, stomatal conductance and intracellular concentration of CO_2_) were evaluated according to the previously published protocols^107,110^. All the physiological analyses were performed on the second fully developed leaves in 5 replications per time-point. WC and RWC parameters were calculated according to the following formulas: WC% = (FW-DW)/FW and RWC% = (FW-DW)/(SW-DW) × 100, where FW was leaf fresh weight, DW was leaf dry weight, and SW was leaf turgid weight. EL parameter was calculated according to the formula: L1/L2 × 100, where L1 is electrolyte leakage of freshly collected leaves and L2 is full electrical conductivity of those leaves disrupted by liquid nitrogen. All the measurements were prepared using Instruments EC215 Conductivity Meter (Hanna Instruments, Leighton Buzzard, UK). Chlorophyll ‘a’ fluorescence parameters were calculated according to measurements performed with the HandyPEA fluorimeter (Hansatech Instruments Ltd., King’s Lynn, England) during midday in 5 replications. Gas exchange parameters were calculated according to measurements done by LI-6400XT Portable Photosynthesis System (LI-COR, Lincoln, USA) with a RGB-0241 chamber [irradiance = 1000 µmol(quanta)/m^2^/s, LED lamps, CO_2_ reference concentration 380 µmol(CO_2_)/mol, the air-flow rate through the assimilation chamber was 400 µmol/s, relative humidity 30% and chamber temperature 20 °C].

### ABA content measurement

ABA content was evaluated using ‘Plant hormone abscisic acid (ABA) Elisa Kit’ (CUSABIO, www.cusabio.com, CSB-E09159Pl). The measurements were performed in 3 biological replicates for each time-point of experiment according to the provided protocol.

### Chloroplast fructose-1,6-bisphosphate aldolase (pFBA) activity

The activity of pFBA in leaves was measured using the modified Sibley–Lehninger method^111,112^. Native chloroplast proteins were extracted according to the previously published method with slight modifications^49,113^. The amount of 1 g of frozen leaf tissue from 3 biological replicates was homogenized and then suspended in 4 ml of chloroplast isolation buffer (CIB) (Sigma) with 0.1% BSA. Samples were filtered through a Sefar nitrex, mesh 100 and centrifuged at 200×*g* at room temperature for 3 min. Supernatant was carefully collected into new tubes and centrifuged again for another 15 min at 900×*g* at room temperature. Obtained pellet was washed twice with 1 ml of CIB solution without BSA. Finally, the chloroplast pellet was dissolved in 500 µl of protein isolation buffer (0.1 M Na2HPO4, 3% Triton X-100) and after vortexing, centrifuged at 21,500×*g* in room temperature for 10 min. The collected supernatant was used to determine pFBA activity. 100 µl of samples were mixed with isolation buffer. In three tubes per sample, 100 µl of 0.06 M fructose-1,6-bisphosphate was mixed with 140 µl of incubation buffer pH 7.4 (0.05 M 2,4,6-trimethylpyridine, 0.08 M hydrazine sulfate, 0.3 mM sodium iodoacetate) and pre-incubated for 10 min at 30 ^◦^C in water bath. One tube contained additionally 300 µl of 10% trichloroacetic acid (TCA) and was treated as a reference. After pre-incubation, 50 µl of chloroplast extract was added, mixed and incubated at 30 °C for 2 h. After that time, the reaction was inhibited by 300 µl of 10% TCA. Ice-chilled samples were spined down. 100 µl of collected supernatant from each tube was mixed with 100 µl of 0.75 M NaOH and incubated at room temperature for 10 min. Further, 100 µl of 0.1% 2,4-dinitrophenylhydrasine was applied to the samples. After the 10 min incubation at 30 °C, 700 µl of 0.75 M NaOH was added and mixed. Absorbance at 540 nm wavelength was measured within the next 10 min. A standard curve was prepared by a sequence of 0.1 mM d-glyceraldehyde dilutions^84^. The activity of pFBA was calculated as the amount of trioses produced by pFBA in 1 g of the fresh sample during 1 h.

### Antioxidant enzymes activity

The activity of ascorbate peroxidase (APX) was measured using Ascorbate Peroxidase Microplate Assay kit (Cohesion Biosciences, CAK1052), according to the manufacturer’s instructions. Finely grinded leaf tissue collected from 3 biological replicates per time-point were used. The amount of enzyme necessary to oxidize 1 µmol of ascorbic acid per minute represented one unit of APX activity.

The activity of catalase (CAT) was determined according to previously published protocol^49,114^ with slight modifications. For this procedure, a leaf extract was prepared from 0.2 g homogenized leaf tissue from 3 biological replicates per time-point. Tissue was homogenized in 1 ml of 50 mM KH_2_PO_4_ buffer, pH 7.0 and centrifuged at 14,000×*g* for 20 min at 4 °C to collect the supernatant. The activity of CAT was determined spectrophotometrically based on the absorbance change at 240 nm on the basis of the amount of decomposed H_2_O_2_. One unit of CAT activity was the amount of enzyme used to catalyze a decomposition of 1 μmol H_2_O_2_ per minute calculated from the extinction coefficient 45.2/mM/cm.

The protein content in the samples was determined by the Bradford’s method^109^. The values of each enzymatic activity measurements were subsequently normalized to the level of soluble protein content in the sample and expressed per mg of protein. Spectrophotometric measurements were performed using the Synergy HTX Multi-Mode Reader (BioTek).

### Superoxide anion radical (O_2_^·−^) and hydrogen peroxide (H_2_O_2_) content

Content of O_2_^·−^ was assayed spectrophotometrically according to the previously described procedures^49,115,116^. Discs of approximately 0.8 cm in diameter were cut out from the second fully expanded leaves and incubated for 1 h in the dark with 3 ml of mixture pH 7.8 (0.05 M KH_2_PO_4_/K_2_HPO_4_, 0.1 mM EDTA, 10 mM NaN_3_ and 0.05% NBT). Nitroblue tetrazolium (NBT) undergoes reduction by O_2_^·−^ and forms colorful diformazan. After the incubation, the samples were heated 15 min at 85 °C. After the cooling down, the absorbance was measured at 580 nm. The amount of O_2_^·−^ was presented as the absorbance level per 1 g of leaf fresh weight (FW).

H_2_O_2_ content was evaluated by the titanium (Ti^4+^) method^49,117,118^. The amount of 0.4 g of plant leaf tissue in 3 biological replicates was homogenized in TissueLyser (Qiagen) with cooled 1.5 ml of 0.1 M potassium-phosphate buffer (pH 7.8) and subsequently centrifuged at 14,000×*g* at 4 °C by 25 min to collect supernatant. A reaction mixture contained 400 µl of tissue extract, 600 µl of potassium-phosphate buffer and 500 µl of titanium reagent (0.6 mM PRL and 0.6 mM PTO in a ratio 1:1) in 2 technical replicates per sample was incubated 10 min at room temperature. After the incubation, the absorbance was measured at 508 nm using Ultrospec 1100 pro spectrophotometer (Amersham). The level of H_2_O_2_ was calculated according to the standard curve and expressed as µmol H_2_O_2_ per 1 g of FW.

### Malondialdehyde (MDA) content

The content of MDA was estimated according to thiobarbituric-reactive substances (TBARS) level in 3 independent samples. TBARS content was measured spectrophotometrically at 532 nm and 600 nm^119^ with slight modifications^49^. The amount of TBARS was calculated according to the formula: TBARS (µM) = (A_532_–A_600_)/155, where 155 is an extinction factor and expressed per 1 g of FW.

### Statistical analysis

All the statistical analyses were performed with the STATISTICA 10.0 software (StatSoft, Tulsa OK, USA). A two-way analyses of variance (ANOVA), with genotype and time-point as classification factors, were performed. Differences between the objects during the experiment duration were evaluated using Fisher’s least significant difference (LSD) test at *p*-value 0.05. Homogeneity groups according to the test were denoted by the same letters on the graphs.

## Data Availability

The data underlying this article are available in the article, the raw data will be shared on reasonable request to the corresponding author.

## Abbreviations

*Pn*: CO_2_ assimilation rate
ABA: Abscisic acid
APX: Ascorbate peroxidase
CAT: Catalase
*Ci*: Intracellular concentration of CO_2_
cPTIO: 2-4-Carboxyphenyl-4,4,5,5-tetramethylimidazoline-1-oxyl-3-oxide
*E*: Transpiration
EL: Electrolyte leakage
*Fv/Fm*: Maximum quantum efficiency of photosystem II
GSNO: Nitrosoglutathione
*g*_*s*_: Stomatal conductance
HDT: High drought tolerant
LDT: Low drought tolerant
MDA: Malondialdehyde
NR: Nitrate reductase
pFBA: Chloroplast fructose-1,6-bisphosphate aldolase
PTIO: 2-Phenyl-4,4,5,5-tetramethylimidazoline-1-oxyl-3-oxide
ROS: Reactive oxygen species
RWC: Relative water content
SNP: Sodium nitroprusside
WC: Water content
